# En bloc resection for treatment of tumor-induced osteomalacia: a case presentation and a systematic review

**DOI:** 10.1186/s12957-015-0589-3

**Published:** 2015-05-08

**Authors:** Tong Meng, Wang Zhou, Bo Li, Huabin Yin, Zhenxi Li, Lei Zhou, Jinhai Kong, Wangjun Yan, Xinghai Yang, Tielong Liu, Dianwen Song, Jianru Xiao

**Affiliations:** Department of Bone Tumor Surgery, Changzheng Hospital, Second Military Medical University, 415 Fengyang Road, Shanghai, China

**Keywords:** Tumor-induced osteomalacia, Phosphaturic mesenchymal tumor, Hypophosphatemia, Thoracic spine, Tumor

## Abstract

**Background:**

Tumor-induced osteomalacia (TIO) is a rare disorder, which is commonly found in craniofacial locations and in the extremities. To the best of our knowledge, only 16 cases have been described in the spine, and this is the first report to describe a case of patient with TIO in the thoracic spine combined with a mesenchymal hamartoma which had confused the therapeutic strategies to date.

**Case description:**

We report the case of a 60-year-old patient with hypophosphatemia and presented with limb weakness. Treating with phosphate did not correct the hypophosphatemia and an ^111^In pentetreotide scintigraphy (octreotide scan) revealed an increased uptake at the right forearm. The tumor was resected totally, and the histopathology revealed a mesenchymal hamartoma, but we noticed that hypophosphatemia was not corrected after the tumor resection. Then a whole-body magnetic resonance imaging (WB-MRI) was performed and the results revealed tumorous tissues at the right T1 vertebral pedicle. The tumor was removed with an en bloc method, and the pathology showed phosphaturic mesenchymal tumor. Follow-up at 1 year after surgery revealed no recurrence, and the serum phosphorus level of the patient was normal.

**Conclusions:**

Tumor-induced osteomalacia is exceedingly rare with only 16 cases in spine published in the literature. It is difficult to find and leads to years of suffering debilitating complications. In this regard, the WB-MRI is a better method to locate the real tumor. Treating with phosphate can only relieve symptoms, and a complete surgical removal remains the gold standard treatment.

**Electronic supplementary material:**

The online version of this article (doi:10.1186/s12957-015-0589-3) contains supplementary material, which is available to authorized users.

## Background

Tumor-induced osteomalacia (TIO), also known as oncogenic osteomalacia, is a rare paraneoplastic syndrome, which is characterized by hyperphosphaturia, hypophosphatemia, and increased levels of alkaline phosphatase [1,2]. Clinical characteristics often include bone pain, pathologic fractures, and musculoskeletal weakness [3], which are related to renal phosphate wasting and resultant reduction in bone mineralization [2,4,5]. TIO was first described in 1947 by McCance and colleagues [6] who reported a 15-year-old girl exhibiting osteomalacia with vitamin D resistance. Unfortunately, he did not attribute her osteomalacia to femoral tumor.

In this report, we illustrate the clinical presentation and therapeutic strategy of a patient with a TIO in thoracic spine. He underwent an en bloc resection to attain oncological and metabolic control. To date, about 300 cases have been described in the world’s literature [7], and only 16 cases were located in the spine [5,8-19]. To our best knowledge, it is the first report to describe a mesenchymal hamartoma which confused the real location of TIO. In this report, we also comprehensively reviewed those cases of TIO in spine published in the English language literature, paying particular attention to the clinical presentation and therapeutic strategy of the reported tumors.

## Case presentation

The patient, a 60-year-old man, first noticed pain in his right foot in 2007. In 2008, he came to the primary hospital presenting with low back pain, gait disturbance, and muscle weakness in the right lower limb. Radiological examination did not reveal any pathologic change in the lumbar vertebrae, whereas a bone scan revealed markedly increased bone uptake at the whole skeleton, and positron emission tomography/computed tomography (PET/CT) revealed mild increased uptake at multiple ribs and right femoral trochanter. No regular treatment was performed. Then the patient’s symptoms were progressively worsened and he became disabled. In 2010, blood examination revealed hypophosphatemia (0.5 mg/dl; normal range 0.98 to 1.62 mg/dl), hypocalcemia (2.17 mmol/L; normal range 2.25 to 2.75 mmol/L), reduced 1,25-dihydroxy vitamin D (10.5 pg/ml; normal range 11.1 to 42.9 pg/ml), and elevated β-C-terminal telopeptide of type I collagen (CTX) (1426 pg/L; normal range 100 to 650 pg/L). Osteomalacia was initially diagnosed and treated with phosphate, calcium, vitamin D, and calcitriol. Although blood examination still revealed hypophosphatemia (0.55 mg/dl), symptoms got better progressively. In 2012, an ^111^In pentetreotide scintigraphy (octreotide scan) was performed, and the result revealed a markedly increased uptake at the right forearm. The tumor was resected totally, and the histopathology revealed a mesenchymal hamartoma, but we noticed that hypophosphatemia wasn’t corrected after the tumor resection. In 2013, the patient suffered paraplegia and then a PET/CT scan was performed. Unfortunately, PET/CT scan did not locate the tumor, and thus, he underwent to a whole-body magnetic resonance imaging (WB-MRI), which revealed tumorous tissues at the right T1 vertebral pedicle.

The 60-year-old man was presented to our spine tumor center for consultation regarding resection of the tumor in the right T1 vertebral pedicle. On presentation to our center, the patient had signs of severe osteomalacia, and the patient’s most outstanding complaints were diffuse bone pain throughout the whole skeleton, limb weakness, hypaesthesia, and disabled walking. Physical examination revealed that motor strength was graded as 3/5 in the upper limbs and 1/5 in the lower limbs. Sensation decreased slightly throughout, with hyporeflexia. The Hoffmann sign and the Babinski sign were negative.

Blood tests revealed hypophosphatemia (0.51 mg/dl), hypocalcemia (2.15 mmol/L), elevated levels of parathyroid hormone (PTH) (72.3 pg/mL; normal range 12 to 65 pg/mL), normal serum alkaline phosphatase (ALP) levels (313 U/L; normal range 115 to 359 U/L), and normal levels of 1,25-dihydroxy vitamin D (21.01 pg/ml).

**Figure 1:**
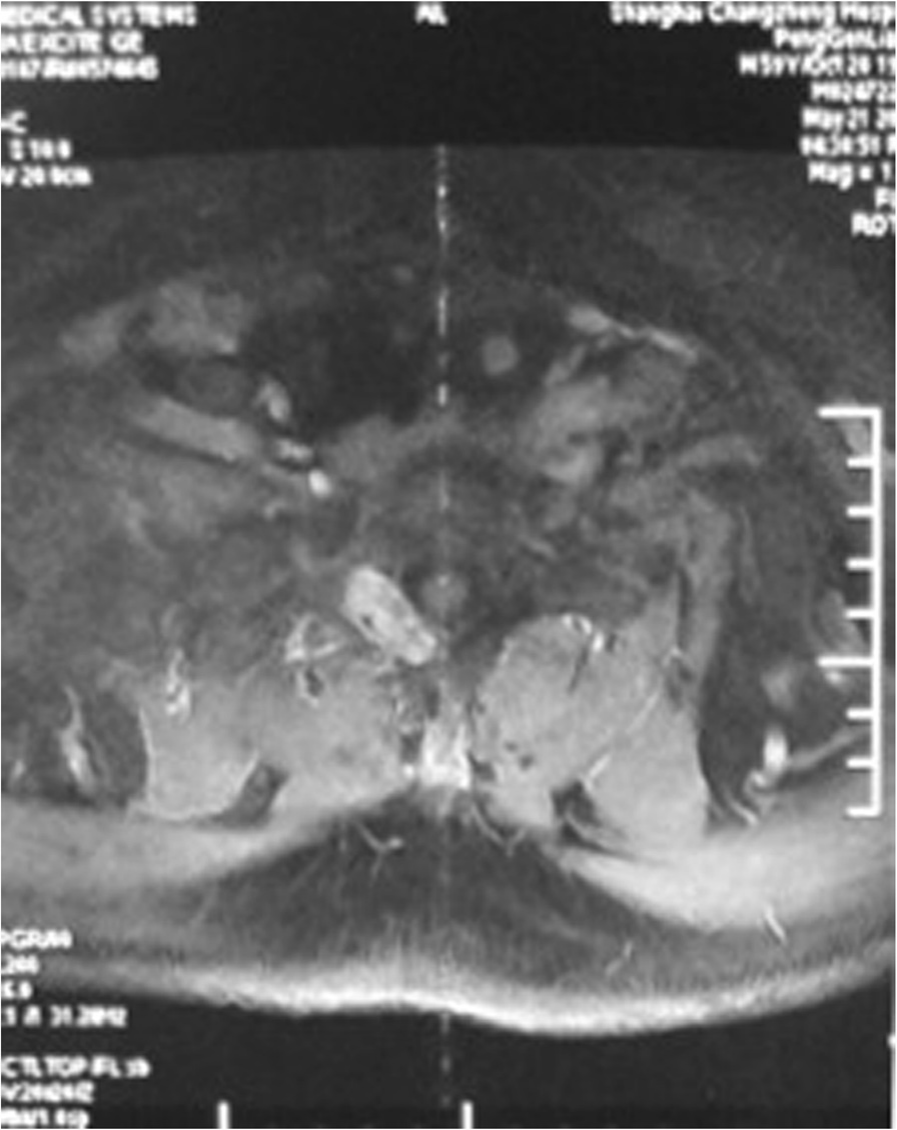
Preoperative gadolinium-enhanced T1-weighted magnetic resonance image (MRI). Axial image shows osteolytic lesion with a relatively smooth margin in the T1 right vertebral pedicle and heterogeneously enhanced with gadolinium.

Radiological exams demonstrated osteolytic changes of the T1 right vertebral pedicle and mild collapse of the T1 vertebral body. In addition, some fractures were detected in multiple ribs. MRI revealed a tumor in the T1 vertebral body and right vertebral pedicle (Figure 1). The tumor showed iso-signal intensity on a T1-weighted image (T1WI), high-signal intensity on T2WI, and was heterogeneously enhanced by gadolinium.

### Surgery

**Figure 2:**
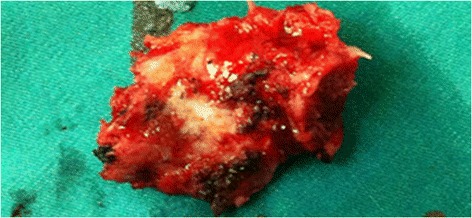
Tumor specimen.

Written informed consent was obtained. We decided to remove the whole metabolically active tissue via an en bloc method, and bilateral vertebral pedicles were used to accomplish spinal reconstruction. Care was taken to resect the tumor around its margins, and cuts were made in the bone outside of the tumor margin to minimize spillage of tumor. A titanium mesh cage was placed in the position of the T1 vertebral body and was packed with recombinant human BMP-2 matrix. A gross total resection was performed without intraoperative complications, and the specimen was sent for pathologic diagnosis (Figure 2).

### Histopathology

**Figure 3:**
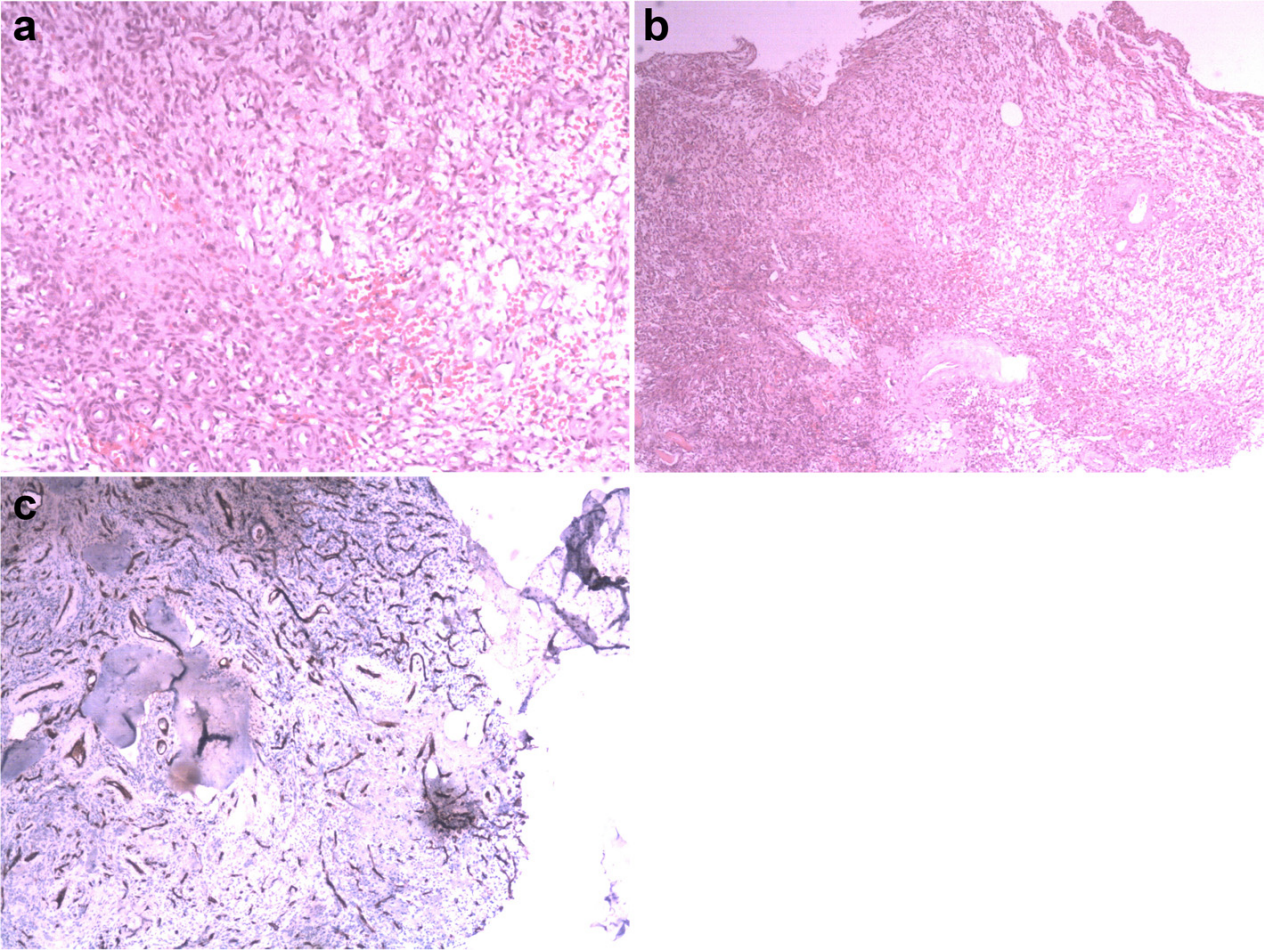
Histopathology of the tumor. **(a)** The tumor mainly comprises short spindle cells with deeply stained nuclei. Few mitoses are observed (Hematoxylin and eosin [H & E]); **(b)** Some areas of vascular proliferation show larger vessels arranged in a ‘staghorn’ pericytoma pattern; **(c)** Immunostaining for CD34 reveals many tumor cells with positive staining in the cytoplasm.

The cells appeared spindled with normochromatic, small nuclei, and indistinct nucleoli. No significant nuclear atypia and mitoses were found. The tumor cells were focally embedded in a myxochondroid and osteoid-like matrix and contained cystic spaces of many sizes, including large dilated spaces filled with blood (Figure 3a). The tumor was well-vascularized, and bone invasion was focally present (Figure 3b). Immunohistochemical staining was positive for CD34 (Figure 3c), Vimentin, and S100, with a quite low proliferation index (Ki-67 1% to 3%). Phosphaturic mesenchymal tumor (PMT) was diagnosed in the patient.

### Follow-up

**Figure 4:**
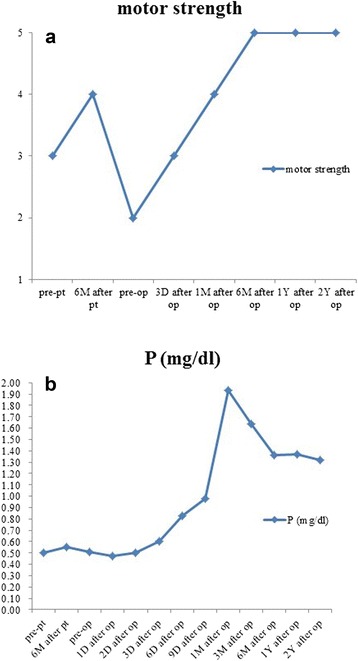
Follow-up. **(a)** Change of motor strength Change; **(b)** Change of serum phosphorus level.

After surgery, the patient stayed in bed for 1 month, and 1.5 μg/day of calcitriol was orally administered. The clumsiness and muscle weakness in both legs of the patient gradually improved, with motor strength graded as 4/5 in the lower limbs at the 6 months follow-up and the grade remained at this level thereafter (Figure 4a). When he walked, he wore a cervicothoracic brace. The patient experienced no multiple bone pain or new neurologic deficits during the 1-year follow-up period. One year after the surgery, the patient had normal neurologic function, normal triceps, biceps knee, and ankle jerks, and he returned to work. Serum phosphorus levels slightly fluctuated, but the levels remained within the normal range (Figure 4b). Bone union without loss of cervicothoracic physiological curvature was confirmed by radiological examination at the 1-year follow-up. No evidence of recurrent tumor was detected in the MRI. The patient had no osteomalacic symptoms, and no new fractures were detected. In addition, we confirmed the accurate bony union of the fractured ribs.

## Discussion

Osteomalacia is a metabolic bone disorder characterized by impaired mineralization of osteoid matrix in mature bone. It is most commonly caused by rare inborn disorders of metabolism, such as inherited hypophosphatemic rickets and X-linked hypophosphatemia (XLH), or may be associated with more common processes such as renal failure [11]. TIO is the most unusual type of osteomalacia, which is commonly found in craniofacial locations and in the extremities [1], along with long-standing hypophosphatemia [1,20,21]. This article presents an unusual case of phosphaturic mesenchymal tumor in the thoracic spine. To our best knowledge, including this one, only 16 TIOs have been described in the English literature in spine (Table 1).

**Table 1:** **Characteristics of spinal cases of TIO**

**Number**	**Authors, year**	**c**	**Age, sex**	**Period (years)**	**symptoms**	**Preoperative lab**	**Treatment**	**Histological diagnosis**	**Outcomes**
1	Boriani et al, 1978 [8]	S1-3	18, M	DNMI	Bone pain, weakness	Hypophosphatemia, elevated ALP	Complete resection, radiotherapy	Osteosarcoma	NOLF
2	Stone et al, 1992 [9]	T3-4	33, F	DNMI	Bone pain, weakness, fractures	Hypophosphatemia	Complete resection, supplementation of vitamin D, phosphate, and calcium	Neuroendocrine tumor	NOLF
3	Yu et al, 1995 [10]	C2	58, F	DNMI	Musculoskeletal pain, weakness	Hypophosphatemia, elevated ALP and PTH	Partial resection, supplementation of vitamin D and phosphate	PMT	PSA
4	Terek et al, 2001 [11]	S1-2	14, M	1+	Bone pain, skeletal abnormalities	Hypophosphatemia, hypocalcemia, low level of vitamin D_3_, elevated ALP	Partial resection, chemotherapy (doxorubicin)	Osteosarcoma	PSA, ALL
5	Dissanayake et al, 2003 [37]	L2	58, M	3+	Musculoskeletal pains, fractures	Hypophosphatemia, low level of vit D_3_, elevated ALP	Complete resection	Haemangiopericytoma	NOLF
6	Folpe et al, 2004 [5]	C1	32, F	4	DNMI	DNMI	Partial resection, radiotherapy	Malignant PMTMCT	PSA recurrence
7	Chua et al, 2008 [12]	T3	34, F	1	Musculoskeletal pain, fatigue	Hypophosphatemia, hypocalcemia, elevated ALP and PTH	Partial resection, supplementation of phosphate and calcitriol	Plasmacytoma	DNMI
8	Sciubba et al, 2009 [13]	T8	56, F	5	Bone pain, weakness, fractures	Hypophosphatemia, low level of vitamin D_3_, elevated ALP and PTH	Complete resection	PMT	NOLF
9	Pirola et al, 2009 [14]	T4	57, M	DNMI	Fractures, paresthesias	Hypophosphatemia, low level of vitamin D_3_, elevated ALP and PTH	Complete resection, supplementation of vitamin D and phosphate	PMT	NOLF
10	Mavrogenis et al, 2010 [15]	S1	42, F	2	Bone pain, paresthesias	Normal	Complete resection	PMTMCT	NOLF
11	Marshall et al, 2010 [16]	T12	55, F	6+	Bone pain; fractures	Hypophosphatemia	Complete resection	PMTMCT	DNMI
12	Akhter et al, 2011 [17]	C5	52, M	1	Fracture	Hypophosphatemia	Complete resection, supplementation of vitamin D, and phosphate	PMTMCT	NOLF
13	Gandhi et al, 2012 [18]	L4	66, F	2,	Bone pain, weakness	Hypophosphatemia, hypocalcemia, low level of vit D_3_, elevated ALP and PTH	Complete resection	PMTMCT	NOLF
14	Nakamura et al, 2014 [19]	C5	72, M	2.5	Weakness	Hypophosphatemia, elevated ALP and PTH	Complete resection, supplementation of phosphate and calcitriol.	PMTMCT	NOLF
15	Puthenveetil et al [38]	T12	61, F	7	Musculoskeletal pain, weakness, fractures	Hypophosphatemia, increased ALP	Complete resection, supplementation of calcium and phosphate	PMTMCT	NOLF
16	Present case	T1	60, M	6	Bone pain, weakness, paresthesia	Hypophosphatemia, hypocalcemia, elevated PTH	Complete resection, supplementation of calcitriol	PMT	NOLF

PMTMCT: phosphaturic mesenchymal tumor mixed connective tissue type; PMT: phosphaturic mesenchymal tumor; DNMI: Did not mention it; PSA: persistent serum abnormalities; ALP: alkaline phosphatase; NOLF: normalization of lab findings; ALL: acute lymphocytic leukemia.

TIO can lead to debilitating complications and years of suffering. The average age of 16 cases is 48.0 (range 14 to 72) years. It occurs in any age, with preponderance around the fifth decades. Characteristically, TIO affects adults without a predilection for gender, with nine female and seven male in all the spinal TIOs. Diffuse bone pain, caused by poor bone mineralization, is the most frequent symptom in these patients [1]. If treated inadequately, severe osteomalacia may lead to progressive myalgias in adults and gait disturbances, growth stunting, and skeletal deformities in children [18].

Notably, one of the most challenging aspects of TIO is to find the tumor. The occult nature of TIO delays its recognition, and in our case, we employed 6 years to identify the real tumor which may attribute to the mesenchymal hamartoma in the same patient complicating the final diagnosis. In a literature review of all the TIOs [7], the average time from onset of symptoms to a confirmed diagnosis often exceeds 2.5 years. Definitive treatment is further delayed by an average of 5 years due to inability to find the underlying tumor due to the characteristics of being small and slow growing and being located in peculiar or atypical sites.

Typical laboratory findings include hyperphosphaturia, elevated ALP, and low serum levels of vitamin D3, which account for 14/15, 10/15, and 5/15 in the literature, respectively [1,22]. Hypophosphatemia is secondary to inhibition of renal phosphorus reabsorption, and the vitamin D synthetic defect blocks the compensatory rise of calcitriol stimulated by the hypophosphatemia [22]. Moreover, fibroblast growth factor-23 (FGF-23), associated with iron in a pathophysiological mechanism of TIO [23,24], is highly expressed in TIO tumors compared with normal tissues [1,20,21].

Radiographically, it presents with bone cortical thickness, osteoporosis, and fracture [25]. Unfortunately, TIO tumors could not be detected by conventional imaging techniques in all 16 cases. The classic detecting method of TIO is ^111^In pentetreotide scintigraphy (octreotide scan), a scanning technique that detects the expression of somatostatin receptors (SSTRs) [26,27]. However, a nonspecific uptake may cause a false-positive scan due to inflammatory tissues, fractures, or other tumor, as lymphocytes can express octreotide receptors, and moreover, a negative octreotide scan cannot exclude a diagnosis of TIO. False-positive scan emphasizes the need to further identify the tumor by PET/CT or WB-MRI exams. Successful tumor localization has also been reported in some patients with PET/CT [28,29]. However, considering the small and occult nature of TIO, it might not be seen as increased uptake of PET/CT utilization [30]. WB-MRI has been well established to have no radiological exposure and offers excellent contrast resolution of bone, soft tissue, and subcutaneous regions. Moreover, majority of TIOs which had been reported to be located in the bone, soft tissue, and subcutaneous region have a small size. Accordingly, WB-MRI is advantageous for detecting such kinds of tumor [31,32].

The ideal treatment for TIO is complete tumor resection, and this corrects the biochemical abnormalities and remineralizes the bone substance in most cases (12/16) [33]. The partial resection (4/16) might also lead to persistent serum abnormalities remained or even tumor recurrence (#6). It is worth noting that osteomalacia may reduce bone resistance and increase the risk of nonunion or lead to delayed union [34]. Therefore, rigid internal fixation and effective brace should be insured [35,36]. With regard to TIO without accurate location, the combination of vitamin D, phosphorus supplementation, and calcitriol can be used to replace progressive renal phosphorus loss, promote renal production of 1,25-dihydroxy vitamin D, and enhance renal phosphorus reabsorption. However, medical therapy cannot maintain long-term efficacy and potential complications should also be watched out, such as hyperparathyroidism, hypercalcemia, and kidney stone formation [1].

## Conclusion

Tumor-induced osteomalacia is exceedingly rare with only 16 cases in spine published in the literature. It is difficult to find and leads to years of suffering debilitating complications. In this regard, WB-MRI is one of the important options to detect the real tumor. Treating with phosphate can only relieve symptoms, and completing surgical removal remains the modality of choice.

## Consent

Written informed consent was obtained from each patient for publication of this study and the accompanying images.
